# Impact of a pre-feeding oral stimulation program on first feed attempt in preterm infants: Double-blind controlled clinical trial

**DOI:** 10.1371/journal.pone.0237915

**Published:** 2020-09-09

**Authors:** Karine da Rosa Pereira, Deborah Salle Levy, Renato S. Procianoy, Rita C. Silveira

**Affiliations:** 1 Department of Speech-Language Pathology, Hospital de Clínicas de Porto Alegre, Porto Alegre, Rio Grande do Sul, Brazil; 2 Department of Health and Communication, Universidade Federal do Rio Grande do Sul, Porto Alegre, Rio Grande do Sul, Brazil; 3 Department of Pediatrics, Universidade Federal do Rio Grande do Sul, Porto Alegre, Rio Grande do Sul, Brazil; 4 Newborn Section, Hospital de Clínicas de Porto Alegre, Porto Alegre, Rio Grande do Sul, Brazil; KU Leuven, BELGIUM

## Abstract

**Objective:**

To evaluate the effect of an oral stimulation program in preterm on the performance in the first oral feeding, oral feeding skills and transition time from tube to total oral intake.

**Study designer:**

Double-blind randomized clinical trial including very preterm newborns. Congenital malformations, intracranial hemorrhage grade III or IV, bronchopulmonary dysplasia, and necrotizing enterocolitis were excluded. Intervention group (GI) received an oral stimulation program of tactile extra-, peri-, and intraoral tactile manipulation once a day for 15 minutes, during a 10-day period. Control group (GII) received sham procedure with same duration of time. Feeding ability was assessed by a speech-language pathologist blinded to group assignment. The classification of infants’ oral performance was determined by Oral Feeding Skills (OFS). Neonates were monitored until hospital discharge.

**Results:**

Seventy-four (37 in each group) were randomized. Mean gestational ages and birth weights were 30±1.4 and 30±1.5 weeks, and 1,452±330g and 1,457±353g for intervention and control groups, respectively. Infants in the intervention group had significantly better rates than infants in the control group on: mean proficiency (PRO) (41.5%±18.3 vs. 19.9%±11.6 (p<0.001)), transfer rate (RT) (2.3 mL/min and 1.1 mL/min (p<0.001)) and overall transfer (OT) (57.2%±19.7 and 35.0%±15.7 (p<0.001)). Median transition time from tube to oral feeding was 4 (3–11) and 8 (7–13) days in intervention and control groups, respectively (p = 0.003). Intake of breast milk was found to reduce transition time from tube feeds to exclusive oral feeding (p<0.001, HR 1.01, 95%CI 1.005–1.019), but the impact of the study intervention remained significant (p = 0.007, HR 1.97, 95%CI 1.2–3.2).

**Conclusion:**

Infants who were breast-fed and an oral stimulation program proved beneficial in reducing transition time from tube feeding to oral feeding.

**Trial registration:**

ClinicalTrials.gov number NCT03025815.

## Introduction

Oral feeding of preterm neonates remains a concern for healthcare providers and parents. It is an essential factor for child growth and development [1]. In preterm newborns, ability to feed orally is one of the criteria for hospital discharge [2]. This population is at high risk of feeding difficulties [3], an estimated 40% of preterm neonates have trouble transitioning from tube feedings (gavage) to oral feeding [4]. Children born before 30 weeks still experience problems at a corrected age of 12 months [5]. Furthermore, improved preterm infant survival has increased the prevalence of feeding problems in children [6].

Achievement of oral feeding is one of the most challenging milestones for preterm infants [7]. Successful oral feeding while still in hospital leads to a rapid transition away from tube feedings; minimizes adverse events such as apnea, bradycardia, and desaturation; and can reduce long-term consequences such as food aversion [8]. Thus, any instrument or therapy that can improve oral skills not only ensures the safety and efficiency of oral feeding, but also shortens length of stay, improves the mother–infant relationship, and saves on hospital costs [9].

Since 2002, when research showed that an oral stimulation program accelerates the transition from tube to oral feeding in preterm neonates, the successful achievement of oral feeding has been viewed from new perspectives [10]. Over the last 16 years, several studies have provided additional evidence of the benefits of this program for early feeding [11], reducing the transition period from tube to oral feeding, shortening length of hospital stay [11, 12], and improving breastfeeding rates [13, 14].

Assessment of oral feeding through the use of performance parameters allows Oral Feeding Skills (OFS) level identification. Oral feeding ability can be classified into four levels as follows: level 1 was defined by PRO < 30% and RT < 1.5 ml/min being the most immature, level 2, by PRO < 30% and RT ≥1.5 ml/min, level 3, by PRO ≥ 30% and RT < 1.5 ml/min, and level 4, the most mature, by PRO ≥ 30% and RT ≥ 1.5 ml/min [15]. Such identification may better demonstrate the effect of the oral stimulation program. Within this context, the present study aims to assess performance in acquisition of oral skills after a program of oral stimulation in preterm infants.

## Materials and methods

This double-blind randomized controlled trial was conducted from August 2015 to November 2016 in a Level III Neonatal Intensive Care Unit (NICU) and a referral center for high risk pregnancies. Our Institution is a baby friendly hospital with an average of 3800 births per years. *Plataforma Brasil* (Number 1.055.594 in May 2015) and Hospital de Clínicas Institutional Research Committee (IRC) (Number 150346 in August 2015) approved this study and each participant or guardian signed an informed consent prior to participation. As the study was considered an internally funded study, the IRC recommended to register subsequently. The authors confirm that all on going and related trials for this intervention are registered in the ClinicalTrials.gov platform–accession number NCT03025815. The study protocol can be accessed at dx.doi.org/10.17504/protocols.io.bcp6ivre.

### Participants

The sample comprised preterm neonates with gestational age of 26 to 32 weeks and 6 days. Neonates with congenital malformations, intracranial hemorrhage grade III or IV, bronchopulmonary dysplasia, and necrotizing enterocolitis were excluded from the study.

### Randomization

Randomization of neonates was performed after written informed consent had been obtained. The randomization process was performed in the Random Alloc Software [16] environment, using a four-block design. First, the sample was stratified for randomization (26–27; 28–29; 30–31; 32). Neonates were, then, randomly allocated, by stratum, into the intervention or control group.

### Procedures

The study intervention was started in the 31st week of postmenstrual age, according to clinical stability.

The intervention group (GI) received an actual oral stimulation program, proposed by Fucile *et al*. [10] whereby the first 12 minutes of stimulation consisted of stroking the cheeks in a circular motion and stroking the vestibular region of the lips, gums, and tongue with the fingertips in an anteroposterior direction. The last 3 minutes of stimulation consisted of nonnutritive sucking. This intervention was also administered throughout 10 consecutive days for blinding purposes. All 74 infants were moved to a separate site, away from parents and staff members, during the intervention/sham periods, 15-30min before tube feeding. All neonates received standard care otherwise.

The control group (GII) received a sham intervention program, which consisted of remaining beside the incubator, for the same time spent in the intervention group, placing the infant in the proper position, and administering gentle perioral touch, without, however, performing the oral stimulation maneuvers themselves. This sham intervention lasted 10 consecutive days. Two speech language pathologists, who were not part of the hospital staff, performed both oral stimulation and sham intervention programs.

### Assessment

Assessment of OFS was performed once the neonate was clinically stable, at 33 weeks or older of postmenstrual age (regardless of weight). The assessment was performed during the first attempt at oral feeding, as prescribed by the attending physician.

All assessments of OFS were performed by the same speech language pathologist, blinded to group allocation. Assessment of oral feeding was performed using a bottle with a slow flow nipple. The specific assessment protocol proposed by Lau and Smith [15] for this study population was used. Neonates’ feeding performance was timed with a stopwatch. During oral feeding assessment, the speech language pathologist filled out a study form designed to collect the following pieces of information: total volume prescribed (ml), total volume taken during feeding (ml), volume taken during the first 5 min of feeding (ml), duration of oral feeding (min), and any episodes of adverse events, such as cough, oxygen desaturation, apnea, and/or bradycardia. Infants were fed for a maximum of 20 min; assessment was early discontinued if adverse events occurred. From the data collected, the following outcomes were calculated: overall transfer (OT, % volume taken/total volume prescribed); proficiency (PRO, % volume taken during the first 5 min/total volume prescribed); and rate of transfer (RT, ml/min). Neonates were classified into four levels of performance according to feeding PRO and RT: level 1, the most immature, was defined by PRO < 30% and RT < 1.5 ml/min; level 2, by PRO < 30% and RT ≥ 1.5 ml/min; level 3, by PRO ≥ 30% and RT < 1.5 ml/min; and level 4, the most mature, by PRO ≥ 30% and RT ≥ 1.5 ml/min. According to Lau and Smith [15], PRO represents nutritive sucking skills during the first 5 minutes while RT reflects fatigue.

Initial and final oxygen saturation, and heart rate were recorded during the feeding assessment. Fatigue was defined as a change in vital signs lasting more than 30 seconds or by the onset of bradycardia or desaturation. Besides vital signs, overall feeding performance, awake stage, sucking-swallowing-respiration coordination, prolonged pauses were also observed to help define fatigue.

Sucking-swallowing-respiration coordination was monitored by observing sucking-swallowing-respiration ratio, pacing, vital signs (saturation, respiratory and cardiac rate) and presence of any respiratory distress during or after feeding.

Fatigue is a non-specific symptom that results from many causes. Fatigue can be associated with changes in infant’s behavior such as the neonate turning his head away from the bottle, making grimaces, presenting prolonged pauses, drooling as well as with modifications in infant’s behavioral state during oral feeding.

These visual perceptions of fatigue are components of our cue-based protocol and they are standardized within our service as part of a routine assessment.

The type of milk ingested, and the number of days from start of oral feeding to achievement of independent oral feeding were also recorded.

### Transition from tube to oral feeding and length of hospital stay

Preterm neonates assessed were recommended to start oral feeds by the % volume (ml) achieved through proficiency (PRO, %ml taken during the first 5 min/ml prescribed). Speech Pathologist was the examiner performing the OFS assessments.

With the initial volume prescribed, dietary progression and transition to full oral feeding was made according to physician’s discretion, based on the cues provided by the infant and on their feeding performance, without interference from speech language pathologist. The volume prescribed to the infant was based on the amount of volume accepted the previous day. Behavioral and physiologic changes during feeding as well as neonate’s feeding performance were observed to guide transition to oral feeding. Physicians and nurses were responsible for monitoring volume acceptance by neonates in each feed every day, 8 oral feedings/day. In our institution, cue-based feeding is considered an important tool to help guide feeding transition. Routinely physicians and nurses gradually increase oral feeds according to neonate’s daily feeding performance. Formula administration via oral route was performed by nursing staff or by the mother herself, who always observed acceptance of oral feeding by the infant. A brief description about the acceptance of each oral diet was carried out by the nursing team on the newborn’s chart. The diet volume was increased gradually (e.g. 5, 10, 20 ml) after medical staff observing, for 24h, the newborns who were able to intake all volume prescribed and did not demonstrate any adverse events.

The duration of transition period was calculated from the date of first oral feeding until gavage tube withdrawal. Length of stay was calculated from NICU admission until the date the neonate was discharged from the hospital.

### Blinding

The parents of participating infants, medical staff, nurses and speech language pathologist who conducted the assessment of OFS were all blinded to allocation. Nurses did not have knowledge about the intervention and control groups; during the intervention period, the nursing staff was informed that participating infants would receive an active or sham oral stimulation intervention, depending on group allocation.

### Breast milk and breastfeeding

After initiating the oral feeding, the type of milk fed to infants was monitored until tube removal. An oral intake form was used to record the timing of oral feeds and whether the neonate had been breast-fed or received formula. The endpoint “breastfeeding at hospital discharge” was considered to have been reached when the infant was discharged with a prescription for breastfeeding.

#### Statistical analysis

The sample size was calculated on the basis of a previous study by Lau and Smith [15], using an estimate of 17% for the level-4 group and considering a relative risk of 3 for neonates in the GI. For a statistical power of 80% and an alpha error rate of 0.05, the sample size was defined as 37 neonates in each group.

All analyses were performed in the PASW Statistics® for Windows, Version 18.0 software environment (Chicago, IL: SPSS Inc. Released 2009). Categorical variables were expressed as absolute and relative frequencies. Symmetrically distributed continuous variables were described as means and standard deviations, while asymmetrical distributed categorical variables were described as medians and interquartile ranges.

Fisher’s exact test was used for comparison of categorical variables. Student’s t test was used for comparisons of symmetrically distributed quantitative variables, while the Mann–Whitney test was used for asymmetrically distributed variables. Bivariate analysis (p < 0.20) was used to ascertain the factors involved. Logistic Regression was used to evaluate the independent association of the factors involved in level 4 (L4) status.

Generalized estimating equations (GEE) were used for analysis of the volume intake in the first days of oral feeding between the groups during the transition period from tube to oral feeding. GEE was carried out to assess volume intake between groups after 10 days of intervention. In the model the factors “day” (post intervention) “group”(intervention and control) and the interaction (days vs group). This analysis was performed to demonstrate in which day the difference in oral volume intake was observed. Finally, the log rank test was used for analysis of the time until transition to full oral feeding. Multivariable Cox proportional hazard model was used to control for breastfeeding rates. The significance level was set at 0.05.

## Results

**Fig 1:**
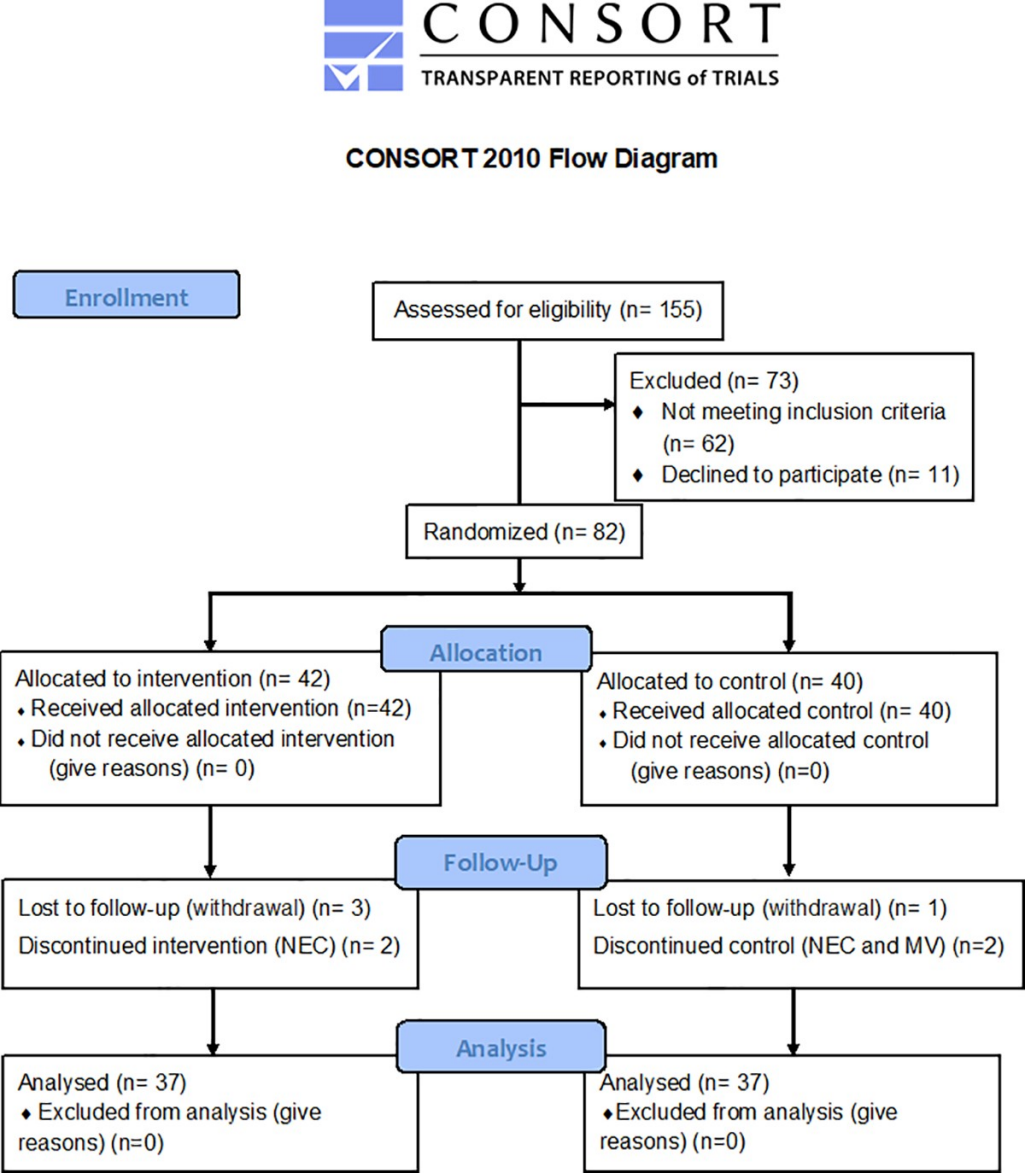
CONSORT flow diagram. Abbreviations: MV Mechanical ventilation, NEC Necrotizing enterocolitis.

During the study period, 1,088 newborns were admitted to the NICU. Of these, 430 were preterm (52 born before 28 weeks, 116 at 29–32 weeks, and 262 at 33–36 weeks). Overall, 155 preterm neonates were considered eligible; 73 were excluded: 62 because their presentation of exclusion criteria (18 bronchopulmonary dysplasia (BPD), 10 BPD and intraventricular hemorrhage (IVH) grades III and IV, 7 IVH grades III and IV, 10 necrotizing enterocolitis (NEC), 6 NEC and BPD or IVH grades III and IV, 3 congenital anomalies and BPD or IVH grades III and IV, 8 congenital anomalies), and 11 because their parents declined to participate; 82 were randomized, and 74 remained in the study until hospital discharge (Fig 1).

The demographic characteristics of the study population are shown in Table 1. There were no statistically significant differences between groups in gestational age, birth weight, 5-minute or 10-minute Apgar score, mechanical ventilation, noninvasive mechanical ventilation, CPAP, small for gestational age, respiratory distress syndrome, or use of CPAP during the intervention period.

**Table 1:** Demographic and clinical characteristics.

Variables	GI (Intervention, n = 37)	GII (Control, n = 37)	P- value
**GA (weeks)**^**a**^	30.7±1.4	30.8 ±1.5	0.608
**26–27** ^**c**^	2 (5.4)	2 (5.4)	1.000
**28–29** ^**c**^	6 (12.6)	6 (12.6)	
**30–31** ^**c**^	17 (45.7)	17 (45.7)	
**32** ^**c**^	12 (32.4)	12 (32.4)	
**Sex, male** ^**c**^	18 (48.6)	19 (51.4)	1.000
**BW (grams)** ^**a**^	1452 ± 330	1457 ± 353	0.954
**Apgar score 5 min** ^**b**^	7 (4–8)	6 (5–8)	0.835
**Apgar score 10 min** ^**b**^	8 (8–9)	8 (7–9)	0.664
**MV** ^**c**^	8 (21.6)	8 (21.6)	1.000
**Duration of MV (h)** ^**b**^	96 (30–144)	120 (40–546)	0.382
**NIMV (h)** ^**c**^	12 (32.4)	12 (32.4)	1.000
**Duration of NIMV (h)** ^**b**^	48 (24–72)	24 (24–90)	0.449
**CPAP** ^**c**^	33 (89.2)	33 (89.2)	1.000
**Duration of CPAP (h)** ^**b**^	48 (24–132)	48 (48–96)	0.636
**SGA** ^**c**^	12 (32.4)	13 (35.1)	1.000
**AGA** ^**c**^	23 (62.2)	24 (64.9)	1.000
**RDS** ^**c**^	24 (64.9)	20 (54.1)	0.478
**CPAP in intervention** ^**c**^	5 (13.5)	2 (5.7)	0.430
**Breastfeeders**			
** Start of oral feeding** ^**c**^	19 (51.4)	16 (43.2)	0.642
** Full oral feeding** ^**c**^	30 (81.1)	31 (83.8)	1.000

GA, gestational age; BW, birth weight; MV, mechanical ventilation; NIMV, noninvasive mechanical ventilation; CPAP, continuous positive airway pressure; SGA, small for gestational age; AGA, adequate for gestational age; RDS, respiratory distress syndrome.

^a^ Student’s t-test (mean and standard deviation)

^b^ Mann-Whitney test (median and interquartile range)

^c^ Fisher’s exact test (frequency and percentage)

Clinical assessment of OFS, mean gestational age was 34.2±0.7 in GI and 34.5±0.9 in GII (p = 0.295). Mean weight at oral feeding assessment was 1875±236 in GI and 1871±296 in GII (p = 0.925). In GI, 6 (16.2%) preterm neonates were classified in OFS Level 1, 3 (8.1%) in OFS Level 2, 28 (75.7%) in OFS Level 4; and in GII, 27 (73.0%) preterm neonates in OFS Level 1, 2 (5.4%) in OFS Level 2, 2 (5.4%) in OFS Level 3, 6 (16.2%) in OFS Level 4 (p<0.001). There was no significant difference in heart rate or oxygen saturation before or after assessment, or between the GI and GII. Caffeine was used more in the GII (27 (73%)) than the GI (15 (40.5%)) (p = 0.009). Table 2 shows comparisons of PRO, RT, and OT between GI and GII.

**Table 2:** Comparison between the two groups in the oral feeding clinical assessment.

	GI (Intervention, n = 37)	GII (Control, n = 37)	P- value
**PRO (%)**^**a**^	41.5 ± 18.3	19.9 ± 11.6	<0.001
**RT (ml/min)**^**b**^	2.3 (1.6–2.9)	1.1 (0.6–1.5)	<0.001
**OT (%)**^**a**^	57.2 ± 19.7	35.0 ± 15.7	<0.001
Initial heart rate^a^	165 ± 9.3	165 ± 9.5	1.000
Final heart rate^a^	168 ± 10.0	171 ± 8.7	0.226
Initial oxygen saturation^b^	97 (96–98)	98 (97–99)	0.265
Final oxygen saturation^b^	97 (96–100)	98 (96–99)	0.952

PRO (proficiency) RT (rate of milk transf) OT (overall transf).

a Student’s t-test (mean and standard deviation)

b Mann-Whitney test (median and interquartile range)

There was also no significant difference in adverse events during oral assessment. Desaturation was observed in 7 (18.9%) infants of each group (p = 1.000). Bradycardia and vomiting were observed in 1 preterm neonate (2.7%) of GII (p = 1.000). Choking was observed in 1 preterm neonate (2.7%) of GI (p = 1.000). Gag reflex was observed in 2 (5.4%) infants of GI (p = 0.493). There were no episodes of apnea, cyanosis, pallor, or hiccups during assessment of oral feeding in either group.

Table 3 illustrates the mean difference of the percentage of volume taken via oral route in the first 8 days after initial assessment (S1 Fig). Day 0 (D0) represents the first day of the intervention, and D11 the first day of oral feeding. Infants in GII were less likely to achieve 100% oral feeding than those in GI, over the same period (p = 0.024).

**Table 3:** Difference between the groups in the volume intake during the first days of oral feeding.

Day	Group	Mean difference	95% CI	P- value^a^
**D11**	Intervention x control	27.54	18.71–36.37	<0.001
**D12**	Intervention x control	31.05	19.85–42.25	<0.001
**D13**	Intervention x control	34.27	22.11–46.42	<0.001
**D14**	Intervention x control	28.01	12.91–43.11	<0.001
**D15**	Intervention x control	24.01	8.69–39.74	0.002
**D16**	Intervention x control	20.81	5.34–36.29	0.008
**D17**	Intervention x control	13.17	-2.88–29.22	0.108
**D18**	Intervention x control	9.82	-6.34–25.99	0.234

a P- values were derived from Generalized estimating equations

The results show that the difference between groups persisted until day 7 of tube-to-oral transition period. For Infants who were breast-fed, the time to transition from tube feeds to exclusive oral feeding was reduced (p<0.001), but the impact of the study intervention remained significant (p = 0.007) (Table 4).

**Table 4:** Cox proportional hazard model results for the outcome time of the transition from tube feeds to exclusive oral feeding.

	HR	95%CI	P- value ^a^
Infants who were breast-fed	1.01	1.005–1.019	<0.001
Impact of the study intervention	1.97	1.2–3.2	0.007

HR, razard ratio

^**a**^ P- values were derived from Multivariable Cox proportional hazard model

Mean gestational age at discharge (weeks) was 36.6±1.6 in GI and 36.8 ±1.6 in GII (p = 0.792), weight at discharge was 2418 ±461 in GI and 2442±519 in GII (p = 0.940). Median length of hospital stay (days) was 20 (20–43) in GI and 32 (25–41) in GII (p = 0.210), transition time to full oral feeding was 4 (3–11) in GI and 8 (7–13) in GII (p = 0.003). Full breastfeeding at discharge was 33 (89.2%) in GI and 34 (91.9%) in GII (p = 1.000), with no statistically significant between-group differences in corrected gestational age at baseline assessment–as shown above.

The factors involved in the performance of infant presenting OFS level 4 are shown in Table 5. Table 6 describes the results of Logistic regression with the factors involved in level 4 classification. The oral stimulation program and the type of feeding tube used during assessment were statistically significant. Caffeine did not interfere with OFS level classification when the logistic regression model was applied (p = 0.871).

**Table 5:** Comparison of factors involved in infant’s Oral Feeding Skills.

Variables	L4 (n = 34)	L1, L2, L3 (n = 40)	P- value
**GA (weeks)** ^**a**^	30.6 ±1.6	30.9 ±1.4	0.290
**BW (grams)** ^**a**^	1443 ±369	1464 ±317	0.793
**Sex, male** ^**c**^	13 (38.2)	24 (60.0)	0.102
**Apgar score 5 min** ^**b**^	6.5 (4–8)	7 (5–8)	0.442
**Apgar score 10 min** ^**b**^	8 (8–9)	8 (7.2–9)	0.941
**MV** ^**c**^	7 (20.6)	9 (22.5)	1.000
**Duration of MV (h)** ^**b**^	96 (40–144)	120 (30–542)	0.886
**NIMV (h)** ^**c**^	11 (32.4)	13 (32.5)	1.000
**Duration of NIMV (h)** ^**b**^	24 (24–48)	24 (24–90)	0.872
**CPAP** ^**c**^	31(91.2)	35 (87.5)	0.719
**Duration of CPAP (h)** ^**b**^	48 (24–132)	48 (48–96)	0.698
**SGA** ^**c**^	11 (32.4)	14 (35.0)	1.000
**AGA** ^**c**^	22 (64.7)	25 (62.5)	1.000
**RDS** ^**c**^	21 (61.8)	23 (57.5)	0.814
**CPAP in intervention** ^**c**^	1 (2.9)	6 (15.0)	0.116
**PMA (weeks) at oral feeding assessment** ^**a**^	34.3±0.8	34.4±0.8	0.938
**BW (weeks) at oral feeding assessment** ^**a**^	1893±238	1856±289	0.555
**Caffeine at oral feeding assessment** ^**c**^	15 (44.1)	27 (67.5)	0.060
**Feeding tube—NG** ^**c**^	17 (50.0)	5 (12.5)	0.001
**Feeding tube—OG** ^**c**^	8 (23.5)	10 (25.0)	0.001
**Feeding tube—OG Kangoroo care** ^**c**^	9 (26.5)	25 (62.5)	0.001
**Intervention** ^**c**^	28 (75.7)	9 (24.3)	<0.001

GA, gestational age; BW, birth weight; MV, mechanical ventilation; NIMV, noninvasive mechanical ventilation; CPAP, continuous positive airway pressure; PMA, postmestrual age; SGA, small for gestational age; AGA, adequate for gestational age; RDS, respiratory distress syndrome, NG, nasogastric tube; OG, orogastric tube.

a Student’s t-test (mean and standard deviation)

b Mann-Whitney test (median and interquartile range)

c Fisher’s exact test (frequency and percentage)

**Table 6:** Factors that influenced the L4 classification of oral feeding ability.

Factors		OR	95% CI	P- value ^a^
**Intervention**	Yes	**74.577**	**11.188–1579.451**	**<0.001**
	No	1		
**Feeding tube**	NG	**30.649**	**30.649–662.408**	**0.004**
	OG	**10.356**	**1.301–224.815**	**0.052**
	OG kangaroo care	1		
**Caffeine**	Yes	0.870	0.145–4.558	0.871
	No	1		
**Sex**	Male	0.533	0.101–2.576	0.435
	Female	1		
**Cpap during intervention**	Yes	**70.290**	**3.558–3867.816**	**0.013**
	No	1		

NG (nasogastric tube) OG (orogastric tube)

a P- values were derived from Logistic Regression (Odds ratio)

## Discussion

This double-blind randomized clinical trial demonstrated that an oral stimulation intervention improves feeding performance at first oral feeding in preterm neonates. Overall, 75.7% infants of GI were on level 4 at oral feeding onset, while only 16.2% infants of GII achieved this level. Measures of PRO, RT, and OT were also significantly superior in GI. Consequently, the GI time to transition from tube feeding to oral feeding was half as long as the GII.

The present study used a novel method to ascertain readiness for oral feeding in preterm neonates, using qualitative and quantitative data for determination of OFS levels. Newborns were evaluated once, by a single trained speech language pathologist, using an evaluation protocol with well-defined parameters. The level of OFS was established at the first oral feeding and it was used to define the volume at which neonates subsequently started on eight daily oral feedings. This contrasts with the design of previous studies in which infants were evaluated repeatedly on several days until an eight-daily feeding schedule was reached [10, 12, 17]. Rocha *et al*. did not report any data on assessment of oral feeding in their double-blind randomized clinical trial [11].

The strength of our study is that, differently of NOMAS [18], which assesses oral-motor skills, we have had an approach to aptitude of first oral feed, as well as to sucking, swallowing, and breathing coordination, involving cognitive aspects.

We found similar results in terms of oral ability level in three studies published by Lau et al. In the first study, the authors evaluated the OFS of preterm neonates aged 26–36 weeks GA with no prior stimulation. Overall, 16.7% preterm neonates achieved the highest level of OFS [15]. In a second study, the authors used the same evaluation protocol to compare very low birth weight infants in two intervention groups (a sucking exercise arm and a swallowing exercise arm) and a control group. Measures of PRO, RT, and OT were superior in the swallowing exercise group than in the other two groups [8]. In the third study, the authors assessed late preterm neonates OFS (34–35 weeks GA) in the first 24 hours of extra uterine life. They found that 5 (30%) 34-week-olds and 19 (60%) 35-week-olds had achieved the highest level of OFS [19].

As in our investigation, Fucile *et al*. found statistically significant between-group differences in percent volume taken and RT during the first oral feeding [12]. Conversely, Lyu *et al*. found no significant difference on the percentage of prescribed volume taken during the first oral feeding. However, their neonates were evaluated using a 5-mL volume, which may have contributed to the low percentage of volume ingested. Furthermore, the absence of blinding and the use of different examiners and different gestational ages may have interfered with their findings [17].

Infant weight at the first OFS assessment was similar in intervention and control groups. This finding is consistent to the literature, and supports the hypothesis that infant weight at the time of assessment is not a determining factor of feeding performance [10–12, 20]. In our study, the oral stimulation program and the type of gavage tube used at the time of feeding were associated with improved oral feeding performance.

Comorbidities negatively affect the achievement of feeding skills as highlighted in previous published paper [21]. In our study we just included stable preterm infants.

Another relevant aspect was that progression of oral feeding was controlled by medical staff. The prescribed dietary volume was based on the volume ingested the day before. The cues provided by each infant were observed to gauge acceptance of oral feeds and, thus, respect the child’s individual rhythm of transition progression to oral feeding. Conversely, in a previous study by Bache *et al*., the number of feeds and volume prescribed were determined by a predefined oral feeding protocol in both groups [14].

Although caffeine administration was more prevalent in the control group, it did not interfere with the feeding performance of preterm neonates classified at lower or higher levels of OFS. This contrasts with a previous study by Jadcherla *et al*., which used a retrospective design and found improved oral feeding performance with increasing gestational age and caffeine use [22].

The transition from tube feeding to full oral feeding was twice as long in the control group. This finding corroborates other results described in the literature, in which the transition from gavage to oral feeding was found to be longer in control groups than in intervention groups [10–12, 17]. Infants in the control group were also less likely to achieve full oral feeding than those in the intervention group, even though it was during the same period. There was no significant between-group difference from day 7 of oral feeding onward.

In the present study, infants who were breast-fed and an oral stimulation program proved beneficial in reducing the time to transition from tube feeding to oral feeding. This novel finding corroborates the results described by Yildiz *et al*., who found that olfactory stimulation with mother’s breast milk reduced the time to transition from tube to oral feeding in preterm neonates [23].

A Cochrane Review has highlighted the benefits of oral stimulation in accelerating transition to full oral feeding, shortening length of hospital stay, and shortening duration of parenteral nutrition as compared to the standard care or non-oral interventions. However, this review did not identify any effect on breastfeeding or weight gain [24]. The present study did not find any difference in breastfeeding rates between the groups. Our facility has been certified by the World Health Organization as a Baby-Friendly Hospital since 1999; hence, our prevalence of breastfeeding was very high, independently of the oral stimulation program.

One possible limitation of the present study is the absence of a significant difference in length of hospital stay between groups. Ability to feed orally is not the only determinant of hospital discharge; other criteria, such as weight and ability to maintain body temperature, are also important [25]. The literature is controversial in this regard: some studies have reported differences, while others found no statistical significance relating length of hospital stay and intervention employed [10, 11, 14, 17]. The present study was designed with OFS level at first oral feeding as the primary outcome, and the sample size was enough to explain the findings. The earlier that parents fed their infants and earlier start of Kangaroo mother care have positively affected the achievement of independent oral feeding [26]. However, we did not study the relationship between the parental involvement and oral affect feeding skills.

The oral stimulation program used in our study effectively encouraged acquisition of OFS in clinically stable preterm neonates, promoting a faster and more effective transition from tube feeding to oral feeding. The intervention group neonates were more likely to achieve full (100%) oral feeding as compared to those in the control group. Again, it must be noted that all neonates who were enrolled in this research were medically stable. Therefore, future studies should be designed to assess the efficacy of an oral stimulation program in sicker preterm neonates.

## Supporting information

S1 Checklist CONSORT 2010 checklist of information to include when reporting a randomised trial*. (DOC)

S1 Fig Difference between the groups in the volume intake during the first days of oral feeding. (TIF)
