# Supplementary figures and images for: Impact of a pre-feeding oral stimulation program on first feed attempt in preterm infants: Double-blind controlled clinical trial
Source: PLoS One. 2020 Sep 9;15(9):e0237915. doi: 10.1371/journal.pone.0237915 (PMC7480839; doi:10.1371/journal.pone.0237915)

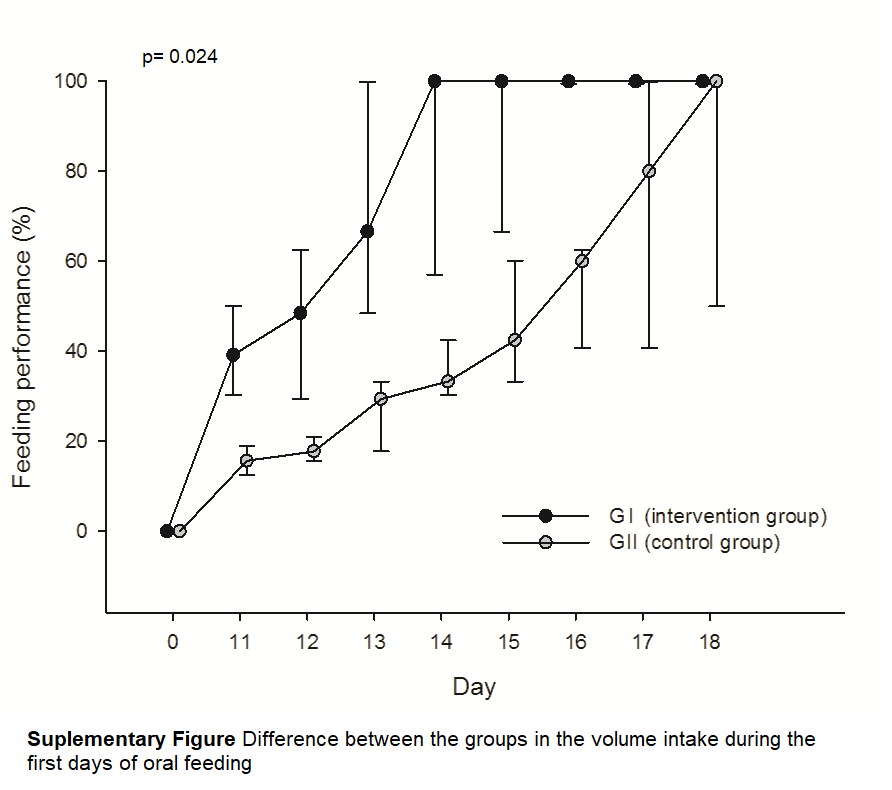

Supplement: S1 Fig — (TIF) [file pone.0237915.s002.tif]
